# Validation of stable reference genes in *Staphylococcus aureus* to study gene expression under photodynamic treatment: a case study of SEB virulence factor analysis

**DOI:** 10.1038/s41598-020-73409-1

**Published:** 2020-10-01

**Authors:** Patrycja Ogonowska, Joanna Nakonieczna

**Affiliations:** Intercollegiate Faculty of Biotechnology, University of Gdansk and Medical University of Gdansk, Abrahama 58, 80-307 Gdansk, Poland

**Keywords:** Microbiology, Molecular biology

## Abstract

Staphylococcal enterotoxin B (SEB), encoded by the *seb* gene, is a virulence factor produced by *Staphylococcus aureus* that is involved mainly in food poisoning and is known to act as an aggravating factor in patients with atopic dermatitis. Research results in animal infection models support the concept that superantigens, including SEB contribute to sepsis and skin and soft tissue infections. In contrast to antibiotics, antimicrobial photodynamic inactivation (aPDI) is a promising method to combat both bacterial cells and virulence factors. The main aims of this research were to (1) select the most stable reference genes under sublethal aPDI treatments and (2) evaluate the impact of aPDI on s*eb*. Two aPDI combinations were applied under sublethal conditions: rose bengal (RB) and green light (λ_max_ = 515 nm) and new methylene blue (NMB) and red light (λ_max_ = 632 nm). The stability of ten candidate reference genes (*16S rRNA, fabD, ftsZ, gmk, gyrB, proC, pyk, rho, rpoB* and *tpiA*) was evaluated upon aPDI using four software packages—BestKeeper, geNorm, NormFinder and RefFinder. Statistical analyses ranked *ftsZ* and *gmk* (RB + green light) and *ftsZ, proC,* and *fabD* (NMB + red light) as the most stable reference genes upon photodynamic treatment. Our studies showed downregulation of *seb* under both aPDI conditions, suggesting that aPDI could decrease the level of virulence factors.

## Introduction

*Staphylococcus aureus* is a gram-positive bacterium that is responsible for certain severe diseases, e.g., scalded skin syndrome, staphylococcal food poisoning or toxic shock syndrome (TSS)^1^. A wide range of staphylococcal virulence factors are implicated in the pathogenesis of diseases caused by this species^2^. The superfamily of *S. aureus* enterotoxins represented by staphylococcal enterotoxins (SEs), staphylococcal enterotoxin-like toxins (SE*l*s) and toxic shock syndrome toxin-1 (TSST-1) contains a significant number of staphylococcal virulence factors^3^. SEs are a family of five serological types of enterotoxins (SEA, SEB, SEC, SED, and SEE) that are stable under high temperature and acidic conditions, which is a very important feature in terms of food safety^1^.

Staphylococcal enterotoxin B (SEB) is one of the most potent SEs. SEB is mainly involved in staphylococcal food poisoning and has been studied for potential use as a biological weapon in an aerosolized form^4^. However, recently, many researchers have underlined the role of SEB in sepsis and in skin and complex soft tissue infections^5^. Additionally, it is known that SEB aggravates inflammation in patients suffering from atopic dermatitis (AD). SEB was shown to act as a superantigen and induce lesions in AD patients^6^.

*Staphylococcus aureus* belongs to the group of pathogens that includes the highly antimicrobial-resistant species *Enterococcus* spp., *S. aureus*, *Klebsiella pneumoniae*, *Acinetobacter baumannii*, *Pseudomonas aeruginosa* and *Enterobacter* spp. and is known as ESKAPE^7^. Due to increasing resistance to antibiotics, alternative therapies to combat the public health risks of *S. aureus* are needed. A promising approach to address antimicrobial resistance is antimicrobial photodynamic inactivation (aPDI), which is effective against viruses, gram-positive bacteria, gram-negative bacteria, fungi and parasites^8^. aPDI is based on the use of a nontoxic dye (photosensitizer, PS), visible light of an appropriate wavelength and oxygen^9^. During the photoinactivation process, two types of possible mechanisms can occur. In the type I reaction, there is electron transfer from the triplet state PS to a substrate, which produces cytotoxic reactive species, such as hydroxyl radical (HO·) or superoxide (O_2_^-•^). The type II reaction involves energy transfer from the PS triplet state to molecular oxygen (triplet ground state) to produce singlet oxygen, which is highly cytotoxic. Both types of reactions produce highly toxic reactive oxygen species (ROS) that target bacterial cell constituents, e.g., proteins, lipids, and nucleic acids^8^.

The primary advantages of aPDI are its localized action and safety for host tissues^10^. Apart from a recent observation that *S. aureus* tolerated treatment with 15 consecutive cycles of passages under sublethal aPDI conditions^11^, no resistance selection for aPDI has been shown thus far. aPDI effectively eradicates a wide group of multidrug-resistant bacteria in vitro (planktonic and biofilm cultures), e.g., vancomycin-resistant *Enterococcus faecalis* (VRE) (5.37 log_10_ unit reduction in survival)^12^, a methicillin-resistant *S. aureus* (MRSA) strain (5–6 log_10_ unit reduction)^13^, and extended-spectrum β-lactamase (ESBL)-producing *K. pneumoniae*. Effective biofilm reduction under aPDI was also documented^14^. A number of confirmatory studies on in vivo*/*ex vivo models have indicated the efficient reduction in viable bacterial cell numbers. A notable example is the application of a blue light-activated porphyrin derivative (TMPyP) or red light-activated phenothiazine chloride (methylene blue, MB) to decrease *E. faecalis* survival in a human tooth model (6.5 log_10_ and 5.8 log_10_ reductions in CFU, respectively)^15^. A white light-activated cationic C_60_ fullerene derivative was applied in an MRSA-infected murine wound model and exhibited a therapeutic effect in the aPDI-treated group after 24 h, observed as a dramatic decrease in the bioluminescence signal^16^.

One of the prominent features of aPDI, in contrast to antibiotic treatment, is the possibility of virulence factor destruction. The activities of V8 protease, alpha-haemolysin and sphingomyelinase were shown to be inhibited in a dose-dependent manner by exposure to laser light in the presence of MB^17^. Blue light, by activating endogenous PSs, reduced the activity of certain quorum-sensing (QS) signalling molecules in *P. aeruginosa*^18^. aPDI may inhibit virulence factors and reduce the in vivo pathogenicity of *Candida albicans*^19^.

The targeting of virulence factors by aPDI, although promising, has only recently started to be more widely explored. Sublethal aPDI using a diode laser and toluidine blue O (TBO), MB and indocyanine green (ICG) decreased the expression of the *fimA* gene, which is involved in biofilm formation in *Porphyromonas gingivalis*^20^. Suppression of the *rcpA* virulence factor gene (3.83-fold reduction) after application of a combination of MB and a diode laser at sublethal doses was documented in *Aggregatibacter actinomycetemcomitans*^21^*.* Additionally, in *S. aureus*, after sublethal aPDI treatment (TBO and a diode laser), a tenfold and 6.2-fold reduction in the *chp* and *shfp* genes, respectively, was demonstrated^22^. Knowledge of whether aPDI at sublethal doses might influence virulence factor production and regulation is currently of great interest and importance. One of the methods used to study this phenomenon is quantitative polymerase chain reaction (qPCR). Unfortunately, very often, the only reference gene used in this type of study is the *16S rRNA* gene, encoding 16S ribosomal RNA, which might not always be the optimal choice. Currently, the problem of selecting a stable reference gene under different experimental conditions is being discussed more extensively. Gene stability depends on the growth phase and metabolic or experimental conditions^23^. Therefore, arbitrary use of an inappropriate reference gene based solely on a literature search may lead to incorrect results and observations^24^. In addition, it is recommended that more than one reference gene be used for high accuracy and reliability of the results^25^.

To avoid inaccuracies, careful selection of reference genes and in-depth analysis of the genes is required. To date, there is no information about the selection of suitable reference genes under photodynamic inactivation. In eukaryotic cells, the *gapdh* gene has been used for routine normalization during quantitative gene expression analysis. In fact, *gapdh* has been used as a historical reference gene for many years, mainly in Northern blotting, RNAse protection assays or conventional qPCRs. Therefore, when real-time PCR techniques started to become more popular, *gapdh* became a natural candidate reference gene. While this gene is a good reference gene under certain conditions, it is completely unsuitable as a reference gene under other conditions. In contrast to eukaryotic cells, in prokaryotic cells, there has been no universal standard gene (similar to *gapdh*) identified due to the high variability of microbial responses to different physiological conditions. Historically, the *16S rRNA* gene has been employed for normalization of gene expression data in bacteria; however, this gene is apparently not universal, and its abundance is too high for many applications, e.g., for studying low levels of mRNA^26^.

Based on available literature data, we chose ten candidate genes to serve as references for qPCR under various conditions. These genes belong to a group of housekeeping genes and basic cellular metabolic processes, such as translation, replication, transcription and cell division. A list of the candidate genes together with an explanation of the process that each gene participates in is provided in Table 2. Based on our analysis, the following genes were selected as stably expressed genes under aPDI: ***ftsZ*** and ***gmk*** for rose bengal (RB) and green light treatment, and ***ftsZ***, ***proC***, and ***fabD*** for new methylene blue (NMB) and red light treatment. In this study, the most stable reference genes under photodynamic treatment were used for measuring enterotoxin gene expression. In addition, the expression levels of *seb* under two photodynamic treatment conditions were employed based on the best selected reference genes.

## Results

### Evaluation of sublethal aPDI conditions

Sublethal conditions of photodynamic treatment were chosen for this study. The goal was to not exceed a value of 0.5 log_10_ reduction in bacterial survival to evaluate the influence of aPDI treatment on bacterial virulence without inactivating the entire bacterial population. The assumption is that when a population of cells is treated, e.g., in an infected wound or lesional skin, not every bacterial cell receives a similar dose of light or PS. Thus, part of the population is subjected to sublethal aPDI treatment. On the other hand, many bacterial populations that coexist in a single niche may respond differently to aPDI due to the presence of various subpopulations that may differ with respect to growth rate, antioxidant enzyme production, PS uptake, etc. In Table 1, bacterial survival at two time points is presented for both types of treatments: (1) RB and green light and (2) NMB and red light. The expected sublethal effect was calculated by subtracting the log_10_ CFU/mL value of treated samples (aPDI) from that of untreated controls (Dark). For aPDI with RB and green light, the reduction in bacterial survival was 0.47 log_10_ units at t20 and t40. For aPDI with NMB and red light, the reduction in cell survival was 0.47 log_10_ (t20) and 0.45 log_10_ (t40) units. Treatment with light alone and incubation of the cells with a PS alone did not affect bacterial survival.

**Table 1:** Bacterial survival under sublethal aPDI conditions.

	Cell survival [log_10_ CFU/mL ± SD]^a^							
	RB + green light				NMB + red light			
	Dark	aPDI	L +	PS +	Dark	aPDI	L +	PS +
20 min after aPDI t20	8.25 ± 0.02	7.78 ± 0.03	8.26 ± 0.05	8.27 ± 0.02	8.29 ± 0.00	7.82 ± 0.12	8.29 ± 0.01	8.29 ± 0.00
40 min after aPDI t40	8.28 ± 0.01	7.81 ± 0.03	8.27 ± 0.03	8.27 ± 0.01	8.30 ± 0.00	7.85 ± 0.13	8.30 ± 0.00	8.29 ± 0.01

The presented values represent the mean of log_10_ CFU/mL ± SD (standard deviation) from three independent biological replicates. **Dark**, untreated control (0 J/cm^2^, 0 µM PS, cells kept in the dark); **aPDI**, treated cells (light + photosensitizer); **L + **, cells treated with light only; **PS + **, cells treated with a photosensitizer only and kept in the dark. RB, rose bengal; NMB, new methylene blue. ^a^the number of bacterial cells transferred into log_10_ CFU/mL.

### Evaluation of real-time PCR efficiency

Ten candidate genes, namely, *16S rRNA, fabD, ftsZ, gmk, gyrB, proC, pyk, rho, rpoB*, and *tpiA*, were evaluated as potential reference genes under aPDI treatment. The specificity of amplification for both candidate reference genes and the target gene (*seb*) was confirmed by melting curve analysis (Supplementary Fig. 2) and gel electrophoresis (Supplementary Fig. 3). To apply the Pfaffl method, the qPCR efficiency is needed. Therefore, standard curves of five-fold serial dilutions of cDNA were prepared (Supplementary Fig. 4). The efficiency of qPCR was calculated based on the formula E = 10^(-1/slope)^ and expressed as a percentage (Table 2). qPCR efficiencies within acceptable limits were observed for *seb* and the following candidate reference genes: *fabD, ftsZ, gmk, gyrB, proC, rho, rpoB,* and *tpiA*. *16S rRNA* and *pyk* were excluded from further analysis due to poor qPCR efficiency and the formation of primer dimers.

**Table 2:** Slope values of the standard curves and qPCR efficiency of each candidate gene and the target gene.

No	Gene	Slope	Efficiency	Efficiency (%)
1	*seb*	− 3.136	2.084	108.4
2	*16S rRNA*	Cp values below 15, efficiency ≪ 90%		
3	*fabD*	− 3.086	2.109	110.9
4	*ftsZ*	− 3.418	1.961	96
5	*gmk*	− 3.150	2.077	107.7
6	*gyrB*	− 3.330	1.997	99.7
7	*proC*	− 3.500	1.931	93.1
8	*pyk*	Formed primer dimers and produced unspecific products		
9	*rho*	− 3.241	2.035	103.5
10	*rpoB*	− 3.221	2.044	104.4
11	*tpiA*	− 3.096	2.104	110.4

### Expression stability analysis by BestKeeper

Out of the ten pairs of primers applied in our search for the best reference gene, eight yielded a single, specific product with acceptable qPCR efficiency (Table 2). The next step in our study was to check the stability of the studied reference gene candidates under our experimental conditions, namely, aPDI based on RB and green light or NMB and red light. In addition, it was of interest to determine whether the same reference gene could be applied to both treatments. We applied four available programs to perform the analysis: BestKeeper^27^, geNorm^25^, NormFinder^25^, and RefFinder^28^. Cp values derived from three independent biological replicates and three technical replicates at each time point (t20 and t40) were included.

BestKeeper software is based on pairwise correlation analyses. In this algorithm, raw Cp values (without any transformation) and real-time PCR efficiency were required. BestKeeper software performs in-depth analysis that focuses mainly on the standard deviation of Cp values (std dev [± Cp]) and the standard deviation of the absolute regulation coefficients (std dev [± x-fold])^27^. Furthermore, the Pearson correlation coefficient (r) and *p*-value were calculated by this algorithm. According to the BestKeeper analysis, the reference gene is stable when it meets the following criteria: (1) std dev (± Cp) should be lower than 1, (2) std dev [± x-fold] should be lower than 2, and (3) r should be close to 1^27^.

**Figure 1:**
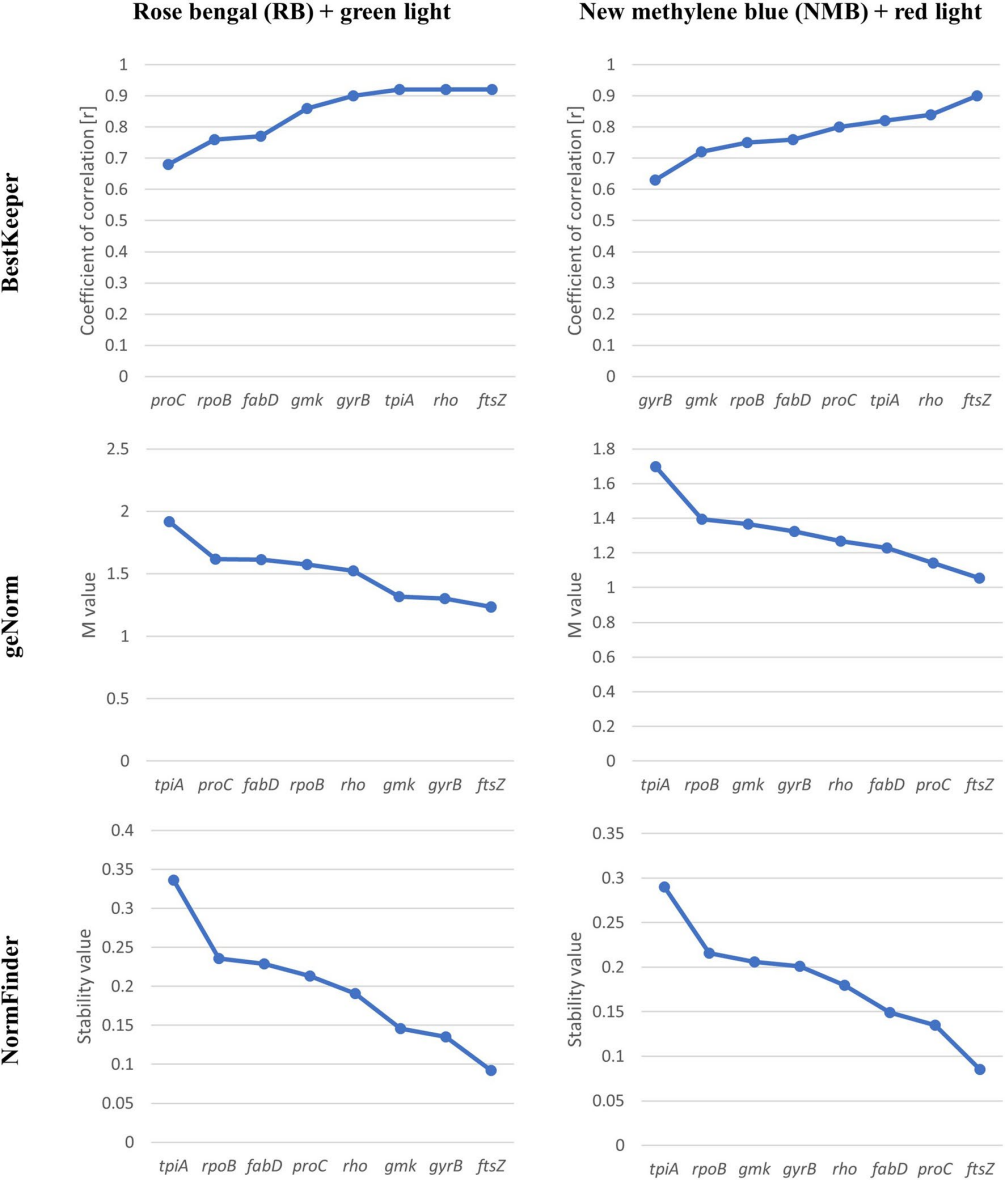
Expression stability ranking of the candidate reference genes according to BestKeeper, geNorm, NormFinder for rose bengal (RB) and green light and for new methylene blue (NMB) and red light. Genes from the analysis were ranked from the least stable (on the left) to the most stable (on the right).

Based on the obtained Cp values, *ftsZ* and *gmk* were indicated as the most stable reference genes upon RB and green light treatment (Table 3). Both *ftsZ* and *gmk* were characterized by the lowest std dev (± CP) (0.87 and 0.88, respectively) and the lowest std dev [± x-fold] values (1.89 and 1.91, respectively). Moreover, the r values were close to 1 (0.92 for *ftsZ* and 0.86 for *gmk*). Other candidate genes also demonstrated r values close to 1; however, the remaining criteria (std dev [± Cp] < 1 and std dev [± x-fold] < 2) were not fulfilled in these cases (Fig. 1; Supplementary Table 1).

**Table 3:** Reference gene stability assessment under aPDI (**RB + green light**) based on BestKeeper analyses.

Gene	aPDI: rose bengal (RB) and green light							
	*fabD*	*ftsZ*	*gmk*	*gyrB*	*proC*	*rho*	*rpoB*	*tpiA*
geo Mean [Cp]	23.67	18.46	17.83	19.36	20.65	21.19	18.28	21.76
min [Cp]	20.50	16.37	15.52	16.43	18.36	17.71	15.32	17.51
max [Cp]	26.14	19.83	19.70	21.83	22.95	25.41	21.43	24.88
std dev [± Cp]	1.43	**0.87**	**0.88**	1.27	1.20	1.63	1.31	2.09
CV [%Cp]	6.04	4.71	4.95	6.52	5.81	7.64	7.16	9.54
min [x-fold]	− 10.50	− 4.07	− 5.45	− 7.61	− 4.50	− 11.93	− 8.28	− 23.49
max [x-fold]	6.25	2.52	3.92	5.55	4.55	20.29	9.42	10.09
std dev [± x-fold]	2.86	**1.89**	**1.91**	2.53	2.41	3.29	2.62	4.62
coeff. of corr.[r]	0.78	**0.92**	**0.86**	**0.90**	0.69	**0.92**	0.76	**0.92**
*p-*value	0.001	0.001	0.001	0.001	0.001	0.001	0.001	0.001

Bold results indicate values that match the criteria.

*[Cp]* crossing point, *geo Mean [Cp]* geometric mean of Cp, *min and max [Cp]* the extreme values of Cp, *std dev [*± *Cp]* standard deviation of Cp, *CV [%Cp]* coefficient of variance of Cp (expressed as percentage, *min and max [x-fold]* the extreme values of expression levels presented as an absolute x-fold over- or under-regulation coefficient, *std dev [*± *x-fold]* standard deviation of the absolute regulation coefficients, *coeff. of corr.[r]* coefficient of correlation between each candidate and the BestKeeper index.

Similar analyses were performed for NMB and red light treatment (Table 4). The results demonstrated that four of the eight tested genes, namely, *proC, fabD, gyrB* and *ftsZ*, met the two criteria of std dev [± Cp] < 1 and std dev [± x-fold] < 2. The *gyrB* gene was excluded because it exhibited the lowest r value (0.63). Therefore, the best reference gene candidates were *ftsZ, proC* and *fabD* (Fig. 1; Supplementary Table 2).

**Table 4:** Reference gene stability assessment under aPDI (**NMB + red light**) based on BestKeeper analyses.

Gene	aPDI: new methylene blue (NMB) and red light							
	*fabD*	*ftsZ*	*gmk*	*gyrB*	*proC*	*rho*	*rpoB*	*tpiA*
geo Mean [Cp]	23.59	18.11	17.96	18.24	20.31	20.64	17.57	21.24
min [Cp]	21.12	16.21	16.10	16.47	19.00	17.32	15.39	17.02
max [Cp]	24.95	19.85	20.68	20.66	21.75	22.61	20.30	24.28
std dev [± Cp]	**0.79**	**0.92**	1.03	**0.86**	**0.78**	1.17	1.12	1.63
CV [%Cp]	3.36	5.08	5.74	4.68	3.83	5.64	6.34	7.66
min [x-fold]	− 6.24	− 3.59	− 3.89	− 3.42	− 2.37	− 10.70	− 4.72	− 22.89
max [x-fold]	2.75	3.23	7.36	5.34	2.58	4.06	7.03	9.54
std dev [± x-fold]	**1.79**	**1.96**	2.13	**1.87**	**1.77**	2.35	2.27	3.31
coeff. of corr.[r]	**0.76**	**0.90**	0.72	0.63	**0.80**	**0.84**	**0.75**	**0.82**
*p-*value	0.001	0.001	0.001	0.001	0.001	0.001	0.001	0.001

Bold results indicate values that match the criteria.

*[Cp]* crossing point, *geo Mean [Cp]* geometric mean of Cp, *min and max [Cp]* the extreme values of Cp, *std dev [*± *Cp]* standard deviation of Cp, *CV [%Cp]* coefficient of variance of Cp (expressed as percentage, *min and max [x-fold]* the extreme values of expression levels presented as an absolute x-fold over- or under-regulation coefficient, *std dev [*± *x-fold]* standard deviation of the absolute regulation coefficients, *coeff. of corr.[r]* coefficient of correlation between each candidate and the BestKeeper index.

### Expression stability analysis based on geNorm and NormFinder

geNorm and NormFinder are popular algorithms that, in contrast to BestKeeper, require normalized Cp values. For this purpose, a relatively simple quantity expressed by the formula M = E^∆Cp^ was used for each reference gene, wherein E corresponds to the qPCR efficiency and ∆Cp corresponds to the lowest Cp value of a studied reference gene minus the Cp value of a particular sample^25^. geNorm was used to calculate gene expression stability using the M value (a measurement of gene stability) based on calculating the pairwise variation in each gene with all other analysed reference genes^25^. A stable reference gene is characterized by an M value that is as low as possible, and the value cannot exceed 1.5. For aPDI treatment with RB and green light, only three reference gene candidates were characterized by M values lower than 1.5: *ftsZ*, *gyrB*, and *gmk*. The least stable gene was *tpiA*, and it was not used in the analysis of gene expression under RB and green light treatment (Fig. 1; Supplementary Table 1). Accordingly, for NMB and red light treatment, four candidate genes exhibited M values lower than 1.5, namely, *ftsZ, proC, fabD,* and *rho*, the last of which exhibited the highest stability. Again, *tpiA* (Fig. 1; Supplementary Table 2) was the least stable gene among those studied under NMB and red light treatment.

NormFinder is used to determine the stability of reference genes, expressed as a stability value. The most stable genes are those with the lowest possible stability values. According to the NormFinder analysis, the most stable reference genes under RB and green light treatment were *ftsZ*, *gyrB*, *gmk* and *rho*, and the least stable candidate was *tpiA* (Fig. 1; Supplementary Table 1). For NMB and red light treatment, the most suitable reference genes for studying expression were *ftsZ*, *proC*, *fabD*, and *rho*. The most unstable gene for NMB and red light treatment was also *tpiA* (Fig. 1; Supplementary Table 2).

Despite the fact that analyses with geNorm and NormFinder were based on different mathematical algorithms, the obtained stability values for the candidate genes were very similar. For RB and green light, four reference genes (*ftsZ, gyrB, gmk,* and *rho*) were similarly ranked in terms of stability by both geNorm and NormFinder. Accordingly, for NMB and red light treatment, the expression of *ftsZ, proC, fabD,* and *rho* was estimated to be stable. Additionally, the algorithms indicated that *tpiA* was unstable under the presented experimental conditions and should not be used.

### Expression stability analysis based on RefFinder

RefFinder is an online software program that ranks candidate reference genes according to their stability under the conditions tested. This ranking is based on the integration of major algorithms—BestKeeper, geNorm and NormFinder—and their rankings. RefFinder assigns a weighted value to each of the studied genes. The final comprehensive ranking is created by calculating the geometric mean of the gene weights. RefFinder differs from other currently available algorithms in that it calculates raw Cp values without considering the efficiency of real-time PCR.

The recommended comprehensive ranking by RefFinder indicated that the most suitable reference genes for expression analysis under RB and green light conditions were *ftsZ, gyrB* and *gmk* (Table 5), which fully correlated with our previous observations.

**Table 5:** Expression stability ranking of the reference genes according to RefFinder for rose bengal and green light.

Method	aPDI: rose bengal (RB) and green light							
	Ranking order^a^							
	1	2	3	4	5	6	7	8
BestKeeper	*gmk*	*ftsZ*	*proC*	*gyrB*	*rpoB*	*fabD*	*rho*	*tpiA*
NormFinder	*ftsZ*	*gyrB*	*gmk*	*rho*	*fabD*	*rpoB*	*proC*	*tpiA*
geNorm	*gyrB/ftsZ*	–	*gmk*	*rpoB*	*rho*	*fabD*	*proC*	*tpiA*
Recommended comprehensive ranking	*ftsZ*	*gyrB*	*gmk*	*rho*	*rpoB*	*proC*	*fabD*	*tpiA*

^a^1, the best among the studied genes; 8, the worst among the studied genes.

Accordingly, *ftsZ, proC, and fabD* were indicated as the best reference genes by RefFinder (Table 6), which confirmed the results obtained by BestKeeper, geNorm and NormFinder.

**Table 6:** Expression stability ranking of the reference genes according to RefFinder software for new methylene blue and red light.

Method	aPDI: new methylene blue (NMB) and red light							
	Ranking order^a^							
	1	2	3	4	5	6	7	8
BestKeeper	*proC*	*gyrB*	*ftsZ*	*fabD*	*gmk*	*rpoB*	*rho*	*tpiA*
NormFinder	*ftsZ*	*proC*	*fabD*	*rho*	*gmk*	*gyrB*	*rpoB*	*tpiA*
geNorm	*ftsZ/proC*	*–*	*rho*	*gyrB*	*fabD*	*rpoB*	*gmk*	*tpiA*
Recommended comprehensive ranking	*ftsZ*	*proC*	*fabD*	*gyrB*	*rho*	*gmk*	*rpoB*	*tpiA*

^a^1, the best among the studied genes; 8, the worst among the studied genes.

### Changes in the expression level of the *seb* gene under aPDI

In the next step, we focused on studying *seb* expression under aPDI after selection of the optimal reference genes. It is recommended that expression data be normalized with respect to two or more reference genes^25^. To normalize the relative expression levels of *seb*, the most stable reference genes were used: *ftsZ* and *gmk* for RB and green light treatment and *ftsZ, proC* and *fabD* for NMB and red light treatment. This approach improved the reliability and accuracy of the measurements. Untreated bacterial cells (Dark) were used as a reference.

**Figure 2:**
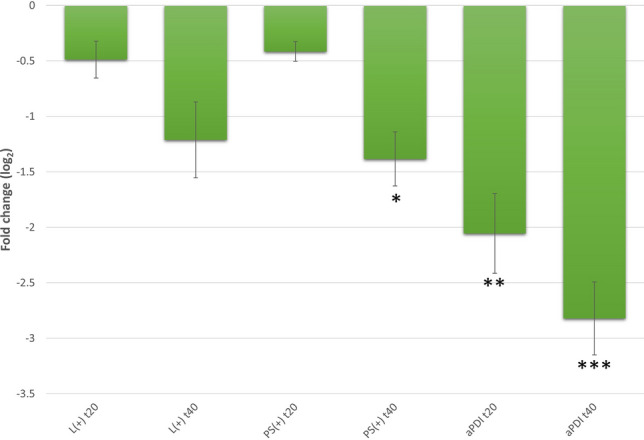
Relative expression of the *seb* gene under aPDI treatment with rose bengal (RB) and green light (λ_max_ = 515 nm). For normalization of the data, the geometric mean of the two most stable reference genes under the tested aPDI conditions (*gmk* and *ftsZ*) was used. Error bars represent the SEM (standard error of the mean) values. Significance at the respective *p-*values is marked with asterisks [*p < 0.05; **p < 0.01; ***p < 0.001 with respect to untreated samples (cells kept in dark)]. L( +), bacterial cells treated with light alone; aPDI, cells treated with RB and light; PS( +), cells treated with RB alone and stored in the dark; t20 and t40, the time points after the irradiation process at which samples were collected.

The results from bacterial cells exposed to RB and green light-mediated aPDI revealed significant downregulation of *seb*. A 2.056 log_2_ unit decrease in *seb* expression after 20 min and a 2.821 log_2_ unit decrease after 40 min of aPDI treatment were observed. These data indicated that the downregulation of *seb* changed over time. In addition, exposing *S. aureus* cells to light alone (L +) also led to downregulation of *seb*, although to a much lesser extent (reductions of 0.489 ± 0.166 log_2_ units after 20 min and 1.212 ± 0.342 log_2_ units after 40 min) than with aPDI. Similarly, RB alone had an impact on the *seb* expression level but only after prolonged incubation (40 min: 1.384 ± 0.244 log_2_ units) (Fig. 2).

**Figure 3:**
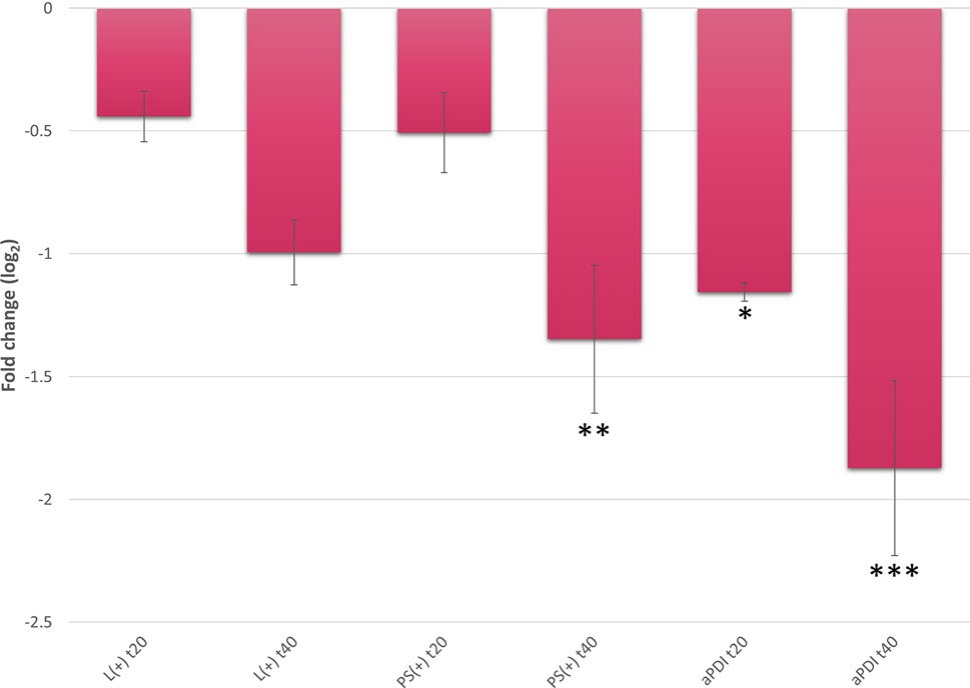
Relative expression of the *seb* gene under aPDI treatment with new methylene blue (NMB) and red light (λ_max_ = 632 nm). For normalization of the data, the geometric mean of the three most stable reference genes under the tested conditions (*fabD, ftsZ* and *proC*) was used. Error bars represent the SEM (standard error of the mean) values. Significance at the respective *p-*values is marked with asterisks [*p < 0.05; **p < 0.01; ***p < 0.001 with respect to untreated samples (cells kept in dark)]. L(+), bacterial cells treated with light alone; aPDI, cells treated with NMB and light; PS(+), cells treated with NMB alone and stored in the dark; t20 and t40, the time points after the irradiation process at which samples were collected.

Accordingly, sublethal aPDI with NMB and red light treatment caused a decrease in *seb* gene expression, similar to RB and green light treatment. At 20 min after irradiation, there was a 1.157 log_2_ unit decrease in *seb* gene expression, and after 40 min, a 1.873 log_2_ unit decrease in the *seb* gene expression level was observed. Treatment with light alone led to a downregulation of *seb*, but these results were not statistically significant (0.441 log_2_ units ± 0.103 for 20 min, *p* > 0.05; 0.995 log_2_ units ± 0.133 for 40 min, *p* > 0.05). In this case, prolonged incubation with NMB without light exposure resulted in decreased *seb* expression (Fig. 3).

### aPDI does not change the antibiotic susceptibility profile

To address the important issue of possible mutation production under aPDI that might result in changes in the antibiotic susceptibility profile, we performed an experiment in which *S. aureus* cells were subjected to 15 consecutive cycles of aPDI treatment. Cells were subjected to sublethal doses of aPDI every other day according to a procedure described in the “Materials and methods”. Following aPDI treatment, cells were washed with PBS, and the entire pool of treated bacteria was used as inoculum for the following culture. Using the entire treated population for the next cycle decreased the probability of missing those cells in which potential mutations could occur. Before the first aPDI treatment (day 0) and after the 1st, 5th, 10th and 15th days of treatment, the antibiotic profile was assessed for all 3 cultures in four parallel experiments by measuring MIC values. The results obtained clearly indicated that after 15 consecutive aPDI treatments, a change in phenotype from antibiotic susceptibility to antibiotic resistance was not observed (Table 7). The same was true for ciprofloxacin treatment, which was used as a control in our experiments. However, for the combination of trimethoprim/sulphamethoxazole, an increased MIC after ciprofloxacin treatment was noticed after the 10th passage that remained after the 15th passage. Interestingly, we observed that the analysed strain that at the beginning of the experiment was resistant to gentamicin (4 µg/mL) became sensitive to this antibiotic (1 µg/mL) after 5 consecutive treatments with RB + green light. This unexpected observation deserves attention and should be investigated in greater detail in the future. However, in neither case was a switch from a sensitive to resistant phenotype observed.

**Table 7:** Antibiotic susceptibility profile of *S. aureus* before and after aPDI.

	MIC value (µg/mL)				
	Day				
	0	1st	5th	10th	15th
**Fusidic acid (FA), S ≤ 1; R > 1**					
Control	0.0625	0.0032	0.125	0.5	0.25
RB + green	0.0625	0.25	0.125	0.25	0.125
NMB + red	0.0625	0.0625	0.0625	0.25	0.5
CIP	0.0625	0.25	0.25	0.125	0.125
**Gentamicin (GEN), S ≤ 1; R > 1**					
Control	4	4	4	4	4
RB + green	4	4	1	1	1
NMB + red	4	4	4	4	4
CIP	4	8	4	4	8
**Linezolid (LZD), S ≤ 4; R > 4**					
Control	0.5	0.5	1	4	2
RB + green	0.5	0.5	0.5	0.5	2
NMB + red	0.5	0.5	1	1	1
CIP	0.5	1	1	1	1
**Mupirocin (MUP), S ≤ 1; R > 256**					
Control	0.25	0.25	0.0625	0.125	0.25
RB + green	0.25	0.125	0.125	0.0625	0.125
NMB + red	0.25	0.125	0.125	0.125	0.125
CIP	0.25	0.25	0.125	0.125	0.125
**Trimethoprim/sulphamethoxazole (SXT), S ≤ 2; R > 4**					
Control	2	2	2	8	4
RB + green	2	2	2	2	2
NMB + red	2	2	2	2	2
CIP	2	2	4	8	8
**Vancomycin (VA), S ≤ 2; R > 2**					
Control	1	1	2	2	2
RB + green	1	1	0.5	0.5	1
NMB + red	1	1	1	1	1
CIP	1	1	1	0.5	1

Control, cells cultured without any treatment; RB + green, cells treated with rose bengal (0.25 µM) and green light (λ_max_ = 515 nm, irradiance 150 mW/cm^2^, total fluence 1 J/cm^2^); NMB + red, cells treated with new methylene blue (5 µM) and red light (red light λ_max_ = 632 nm, irradiance 234 mW/cm^2^, total fluence 16.25 J/cm^2^); CIP, cells treated with ciprofloxacin at sub-MIC concentrations (0.25 µg/mL).

*S* sensitive, *R* resistant, *MIC* minimal inhibitory concentration.

## Discussion

The present research aimed to determine the most stable reference genes in *S. aureus* subjected to aPDI treatment for the study of *seb* gene expression. To date, such studies have not been published.

Stable reference genes were identified to study the expression of the *qacA* and *qacR* genes in *S. aureus* in the presence of antimicrobial compounds. The research group revealed that different sets of genes were stable under berberine treatment (*fabD, proC* and *pyk*) compared to crystal violet or rhodamine 6G treatment (*rho, pyk* and *proC*) and to ethidium treatment (*fabD, tpiA* and *gyrA*). The authors also identified unstable genes in the presence of berberine (*gyrA* and *glyA*), crystal violet (*gmk, gyrA* and *tpiA*)*,* ethidium (*gmk* and *proC*), and rhodamine 6G (*gmk* and *gyrA*) that should be avoided in the analysis^26^. Enterotoxin gene expression in *S. aureus* under osmotic and acidic stress in nutrient-rich medium revealed only four stable reference genes (*rplD, rpoB, gyrB*, and *rho*) out of the nine popularly studied genes^29^. Alternatively, in nutrient-deficient glycerophosphate broth, different genes (*rho* and *proC*) appeared to be the most stably expressed^29^. Thus, it was demonstrated that different genes are stable under different experimental conditions, and careful analysis of reference gene stability should be performed. For determination of stable reference genes and selection of the best candidate for a given experimental condition, BestKeeper, NormFinder and geNorm software could be applied. This approach, however, is often overlooked, and published data are based on known genes that have been used as references under certain conditions but that may not be suitable for other types of conditions. There are no standard genes that can be applied universally in microorganisms, as the expression of typical reference genes may vary greatly under different experimental conditions. The successful use of *gapdh* or *ß-actin* in studies of gene expression in eukaryotes is not always applicable in prokaryotes^30^**.**

One of the most frequently explored reference genes in bacterial gene expression studies is *16S rRNA*. The *16S rRNA* gene has been used in gene expression analysis of several aPDI-treated species in vitro, including *P. gingivalis* and *S. aureus*^22,31^; ex vivo, including *E. faecalis*^32^; and in biofilm cultures, including *E. faecalis, P. aeruginosa* and *Streptococcus mutans*^33–35^. Unfortunately, in the cited references, there are no data on the stability of the selected genes under the studied conditions. Our research showed that the *16S rRNA* gene was the most highly expressed gene and had very low Cp values (Cp values between 7.58 and 8.22), suggesting that it may not be the best reference gene, especially for studying genes with low expression. It has already been documented that the gene encoding the 16S subunit of ribosomal RNA is unsuitable as a reference gene in *K. pneumoniae* and *S. aureus* due to the high abundance of transcripts^26,36^ and the lowest stability under the tested conditions (bacterial growth at 37 °C and 40 °C in the early, middle and late logarithmic growth phases) in *Streptococcus agalactiae*^37^. High expression of the *16S rRNA* gene (Cp value = 10–13) was observed in different phases of growth and under various stress treatments (acid, salt and temperature) in *Corynebacterium glutamicum.* Ranking validated by geNorm revealed that *16S rRNA* was the eighth (growth stages) and seventh (stress treatments) best candidate gene among the thirteen tested. In contrast, analysis conducted by NormFinder indicated that *16S rRNA* was the lowest ranked for both growth phases (eleventh) and stress treatments (tenth)^38^. In addition, different algorithms gave conflicting *16S rRNA* stability results^38^. Subsequently, Krzyżanowska et al*.* proved that the 16S rRNA-coding gene was characterized by poor expression stability in *Ochrobactrum quorumnocens* (Cp value = 10.26) and was one of the most unstable candidate reference genes under 10 different tested culture conditions^39^. The *16S rRNA* gene has also been proven unsuitable for the analysis of iron-regulated gene expression in *Pseudomonas brassicacearum* (it ranked sixth of the eight genes tested)^40^. On the other hand, based on mathematical models (BestKeeper, geNorm and NormFinder software), *16S rRNA* was classified as one of the best reference genes to evaluate expression levels in *Rhodococcus opacus* under different growth conditions^41^. It should be noted that *16S rRNA* may not be suitable for analyses in which the ability to detect nonviable and dead bacterial cells interferes with results^42^. Therefore, we encourage and recommend validation of reference genes with respect to particular conditions studied. The choice of candidate reference genes may be based on, but is not limited to, housekeeping genes.

Three different statistical software packages, namely, BestKeeper, geNorm and NormFinder, were used to assess the stability of candidate reference genes in our experimental setup, namely, photodynamic inactivation of *S. aureus*. The analysis conducted by each software indicated compatible results. For studies on the expression level under RB and green light treatment, the most stable genes were *ftsZ* and *gmk* (BestKeeper), *ftsZ, gyrB,* and *gmk* (geNorm), and *ftsZ, gyrB,* and *gmk* (NormFinder). However, under NMB and red light treatment, the most stable reference genes were *ftsZ, proC,* and *fabD*, as indicated by the 3 software packages. Identical observations were demonstrated by DeLorenzo and Moon^41^. Unfortunately, according to the literature, there were some discrepancies in the results obtained by BestKeeper, geNorm and NormFinder software^36,39,40^. It should be emphasized that such calculation differences may be a result of these three programs being based on different algorithms. Furthermore, for a gene to be classified as stable by BestKeeper, it must meet three basic criteria: (1) std dev [± Cp] should be lower than 1, (2) std dev [± x-fold] should be lower than 2, and (3) r should be close to 1^27^. Unfortunately, the first two criteria are often overlooked. Recommended comprehensive ranking conducted by RefFinder revealed that our obtained results fully correlated with the data from BestKeeper, geNorm and NormFinder for the aPDI treatment under both RB + green light and NMB + red light conditions. Gomes et al*.* reported different observations in which slight differences in results among BestKeeper, NormFinder, geNorm and RefFinder analyses were observed^36^. These discrepancies were mainly observed because RefFinder does not consider the efficiency of qPCR and should not be used as the sole source of data analysis, as this may lead to incorrect observations and false results and conclusions.

The present research proved that different candidate reference genes were stable in the two studied combinations for aPDI; for RB and green light treatment, the most stable reference genes were *ftsZ* (cell division protein) and *gmk* (nucleotide metabolism); for NMB and red light, the most stable reference genes were *ftsZ, proC* (amino acid biosynthesis)*,* and *fabD* (fatty acid biosynthesis). Our observations were in line with those published by Freire et al*.*, in which other reference genes were stable in various photodynamic inactivation experiments in *C. albicans*^43^. This means that the stability of reference genes is strongly dependent on the experimental conditions and thus should be carefully analysed for specific conditions.

Our studies proved that aPDI treatment under sublethal conditions (similar to RB + green light and NMB + red light) can lead to downregulation of the *seb* gene in the *S. aureus* strain tested. It is hypothesized that aPDI, in contrast to classic antibiotic treatment, can effectively influence virulence factor production^44^. This phenomenon has only recently received more attention. Hendiani et al*.* studied the impact of aPDI on QS-controlled genes involved in biofilm formation in *P. aeruginosa*. The research group investigated virulence genes: *rhl* and *las* (QS operons) and *pelF* and *pslA* (biofilm formation). The application of a sublethal dose of MB and a diode laser (λ_max =_ 650 nm) led to downregulation of the expression of the studied genes^34^. Additionally, Fekrirad et al*.* studied the impact of sublethal and lethal aPDI treatment on QS-mediated virulence factors in *Serratia marcescens*. The combination of MB and LEDs (λ_max_ = 660 nm) downregulated the expression of genes necessary for biofilm formation (*bsmA* and *bsmB*), attachment (*fimA a*nd *fimC*), motility (*flhD*) and QS regulation (*swrR*) in *S. marcescens* strains^45^. The downregulation of genes related to adherence (*als3* and *hwp1*), morphogenesis (*cph1* and *efg1*) and biofilm formation (*bcr1 and tec1*) in *C. albicans* after aPDI treatment (MB with a red laser and erythrosine with an LED emitting green light) was demonstrated^43^. These observations confirmed the hypothesis that aPDI could effectively downregulate the expression of several virulence factors in various microbial species.

In the case of *S. aureus*, the possibility of inactivating virulence factors can be of great importance for the treatment of many diseases in which virulence factors may be involved or be a cause. Even if bacteria are not present, virulence factors can be toxic to the host or the environment (e.g., in food poisoning). The in vitro studies conducted so far have demonstrated that the V8 protease, alpha-haemolysin and sphingomyelinase levels were reduced under aPDI (TBO and red laser) in a TBO-dependent manner^17^. Our previous research showed that *S. aureus* supernatants containing aPDI-treated extracellular virulence factors were significantly less toxic to eukaryotic cells than non-treated supernatants^46^. The levels of SEs A and C have been demonstrated to decrease after treatment with light-activated cationic porphyrin^47^. Recently, it was shown that the amount of SEB protein, measured by the western blot technique, decreased after aPDI treatment with TBO and red light (630 nm, 50 mW/cm^2^)^48^. Thus, the potential of aPDI in the destruction of virulence factors appears to be very encouraging. All the presented data are based on in vitro analysis and need to be validated in in vivo models of particular diseases. Our research is the first to investigate the effect of aPDI on SEs at the transcription level. However, the concept of regulating the production of virulence factors with respect to the presence or absence of light has not been widely studied thus far, particularly in chemotrophs, such as *S. aureus*. In this respect, the study of gene (*seb* in our case) expression under sublethal doses of aPDI has great research value.

While applying sublethal doses of aPDI, we have to not only ensure suitable aPDI treatment, i.e., the availability of a proper aPDI dose (PS concentration plus light dose), but also consider situations where many subpopulations of bacteria coexist within a single niche that may respond differently to aPDI. From the previous studies, we could tell that there were several bacterial features that influence the response to aPDI, e.g., superoxide dismutase activity^49^, membrane fluidity^50^, PS uptake^51^, and growth rate^52^. Some cells are exposed to sublethal doses due to insufficient availability of PS or light, whereas others cope better (or worse) due to all the intrinsic features mentioned above.

The light-dependent regulation of gene expression in microorganisms that use light to produce energy (phototrophs) is relatively well known. In contrast, in species representing chemotrophs (e.g., *S. aureus*), cellular processes regulated by light have been less well investigated. In chemotrophic bacteria, light also influences physiology, social life (biofilm), and general behaviour, as the presence of photosensitive proteins has been demonstrated in many representative species, although functional and biochemical analyses are only available for some of these species^53^. Light-sensing proteins in microorganisms have cofactors capable of absorbing light (e.g., flavin adenine dinucleotide (FAD), vitamin B12). These cofactors are structurally similar to many of the chemically synthesized PSs used in aPDI. Therefore, it would be expected that the processes regulated by photosensitive proteins might be disturbed by exogenously added photosensitizing compounds. It can be speculated that photosensitizing compounds used at sublethal doses may affect the transduction of the light signal, ultimately resulting in a specific effect, such as a change in gene expression. The outcome of such light-dependent regulation has been shown to influence a variety of cellular responses^54,55^. In summary, sublethal doses of light or aPDI interfere with intracellular crosstalk; therefore, studying the effect of such doses on living cells is an important safety concern and may contribute to the development of an antibacterial or antivirulence strategy.

Mutation production is a critical safety issue associated with any treatment, including aPDI. aPDI-resistant mutants have not been found. One might expect, however, that ROS produced as a result of aPDI treatment may cause DNA damage. This is possible, but only if the localization of the PS in cells is in close proximity to the DNA, so that short-lived ROS can produce lesions. Data are available to analyse the influence of aPDI on antibiotic susceptibility/resistance profiles in microorganisms. This phenomenon has been studied to some extent by members of our group and others in vitro^56,57^ and in vivo^58^. In none of the cases described so far has an increased resistance to antibiotics been documented. However, it should be remembered that PSs differ in their mode of action, biophysical properties, and localization in the cell and thus in the damage that they cause to the relevant cell biomolecules, including DNA. Therefore, each tested PS should be individually checked for the possibility of causing DNA damage. In our experiment involving 15 consecutive aPDI cycles, no change in phenotype antibiotic susceptibility to antibiotic resistance was observed, indicating that aPDI meets the safety criteria in this particular respect, at least for the PS and light doses used in our studies.

## Conclusions

This study provides an in-depth analysis of the stability of reference genes under photodynamic treatment for the first time. Based on four algorithmic analyses, we recommend using the *ftsZ* and *gmk* genes as stable reference genes for the study of *S. aureus* gene expression (represented here by *seb*) under photodynamic treatment using RB and green light. In addition, the *ftsZ*, *proC* and *fabD* genes are the most stable for studying the expression of various genes in *S. aureus* upon treatment with NMB and red light. The *ftsZ* gene is universal in that it can be used as a reference gene for both green and red light treatments. Finally, we have shown here that upon photodynamic treatment, the expression of *seb* was significantly downregulated after sublethal aPDI, suggesting that the level of this virulence factor may be reduced under the influence of aPDI.

## Material and methods

### Bacterial strain and growth conditions

The reference *S. aureus* strain 140/05 used in the study had a confirmed *seb* toxin gene and was kindly provided by Dr Joanna Empel, National Medicines Institute (NMI), Warsaw, Poland. This strain was characterized by its genetic background and the presence of other enterotoxin genes (Table 8).

**Table 8:** Genetic characterization of the *S. aureus* strain used in this study.

NMI collection number	Phenotype	Clone	*spa* type	CC	*agr*	Toxin genes
140/05	MSSA	CA	t529	CC59	1	*seb*, *selk*, *selq*

Bacterial glycerol stock (25% glycerol) was kept at -80 °C. *S. aureus* was streaked on trypticase soy agar (TSA) (bioMérieux, France), or liquid cultures were grown aerobically in trypticase soy broth (TSB) (bioMérieux, France) at 37 °C in an incubator shaker at 150 rpm (New Brunswick Scientific, Sweden).

### Photodynamic inactivation

#### Chemicals

NMB (dichlorozinc;ethyl-[7-(ethylamino)-2,8-dimethylphenothiazin-3-ylidene]azanium;dichloride) and RB (4,5,6,7-tetrachloro-2′,4′,5′,7′-tetraiodofluorescein disodium salt; Sigma-Aldrich, Germany) were used as PSs in this study. PS stock solutions (1 mM) were prepared in sterile Milli-Q water and stored in the dark at -20 °C. Before use, stock solutions were thawed and diluted in sterile Milli-Q water to an appropriate concentration. PS solutions were stored in the dark at 4 °C for up to a month.

#### Light source

Photodynamic inactivation experiments were carried out using light-emitting diode (LED)-based lamps (SecureMedia, Poland) emitting (1) green light (λ_max_ = 515 nm; irradiance, 150 mW/cm^2^) and (2) red light (λ_max_ = 632 nm; irradiance, 234 mW/cm^2^)^59^. The illumination time for green light was 66 s (total fluence: 2 J/cm^2^), and that for red light was 1709s (total fluence: 20 J/cm^2^). These parameters were based on our initial experiments, in which we screened for the sublethal dose for aPDI, i.e., the dose at which the reduction in cell survival did not exceed 0.5 log_10_ units (Supplementary Fig. 1).

#### Photoinactivation experiment

Overnight *S. aureus* cultures were prepared by inoculating a single bacterial colony in 5 mL of TSB. Overnight cultures were reinoculated in flasks at a ratio of 1:100 and cultured with shaking until the logarithmic growth phase was reached (~ 2–2.5 h, OD_600_ = 0.5). Next, 510-µL aliquots of the *S. aureus* cultures were transferred into 24-well plates. A 5.1-µL aliquot of PS was added to the bacterial cultures at a final concentration of 0.25 µM in the case of RB or 5 µM in the case of NMB. Samples were covered with aluminium foil to protect them from light and incubated for 10 min (RB) or 15 min (NMB) in an incubator shaker (37 °C, 150 rpm). After incubation, the 24-well plates were placed under an LED lamp and irradiated at the appropriate fluence rate. Four combinations of samples were analysed: (1) reference bacterial cells stored in the dark (**Dark**), (2) cells incubated with a photosensitizer and kept in the dark (**PS +**), (3) cells treated with light but with no added PS (**L +**), and (4) cells treated with both light and a PS, i.e., photodynamic inactivation of bacterial cells (**aPDI**). After illumination, *S. aureus* samples were kept at 37 °C in the dark and were collected at two time points, namely, 20 (t20) and 40 (t40) min after irradiation. A 10-µL aliquot of each bacterial sample was transferred into sterile phosphate-buffered saline (PBS) to perform ten-fold serial dilutions (10^–1^ to 10^–4^ in a 96-well plate). A 10-µL aliquot of each dilution was streaked horizontally onto TSA plates and incubated for 18 h at 37 °C to observe bacterial growth. Next, bacterial colonies were counted to assess the number of surviving cells and conditions of sublethal photodynamic inactivation. A reduction in cell number not exceeding 0.5 log_10_ CFU/mL was set as sublethal photodynamic inactivation of bacterial cells. The remaining 500 µL was suspended in 1 mL of the RNA-stabilizing reagent RNA*later* (Sigma-Aldrich, Germany), kept overnight at 37 °C (according to the manufacturer’s instructions) and further subjected to RNA isolation. Each experiment was performed as three independent biological trials.

### RNA isolation

RNA was isolated from *S. aureus* samples using the Syngen Blood/Cell RNA Mini Kit (Syngen, Poland) according to the manufacturer’s instructions with slight modifications. In the first step, the sample suspended in RNA*later* (Sigma-Aldrich, Germany) was centrifuged (6000 rpm and 10 min at room temperature), the supernatant was discarded, and the bacterial pellet was resuspended in 100 µL of bacterial lysis buffer (20 mM Tris–HCl at pH 8.0, 2 mM EDTA at pH 8.0, and 1.2% Triton X-100) with 2 U of lysostaphin (A & A Biotechnology, Poland). The sample was vortexed for 20 s at maximal speed and transferred to a thermal block (37 °C for 30 min). Every 10 min during incubation, the sample was vortexed and centrifuged briefly (15–20 s). Subsequent steps were carried out in accordance with the manufacturer’s instructions. To remove any genomic DNA contamination in the analysed samples, two steps of on-column DNase I digestion were performed (RNase-Free DNase Set, Qiagen, The Netherlands). This step was critical for obtaining pure RNA samples devoid of any genomic DNA contamination. The RNA samples were eluted in 50 µL of RNase-free water. The samples were aliquoted and stored at − 80 °C for further analysis. The quality and quantity of the RNA samples were analysed spectrophotometrically using a NanoDrop 1000 (Thermo Scientific, USA) and with 1.5% agarose gel electrophoresis to confirm the lack of degradation. The electrophoretic separation results were evaluated under UV light (ChemiDoc, Bio-Rad, USA). If only two characteristic bands corresponding to the 16S and 23S RNA subunits were observed, the RNA was processed further.

### Reverse transcription

To transcribe RNA to complementary DNA (cDNA), a TranScriba kit (A & A Biotechnology, Poland) was used. One hundred nanograms of RNA was reverse transcribed with 1 µL of dN-hexamer in a total reaction volume of 20 µL according to the manufacturer’s instructions. The cDNA synthesis conditions were as follows: pre-incubation for 5 min at 25 °C, elongation of hexamers for 60 min at 42 °C, termination for 5 min at 70 °C and cooling at 4 °C. The cDNA samples were stored at − 20 °C for later use.

### Selection of candidate reference genes

Candidate reference genes were selected by searching the literature for studies in which the use of these genes had already been verified under various conditions. The candidate genes represent evolutionarily conserved basic metabolic processes in cells (Table 9). The primers for the analysed genes are summarized in Table 2. The specificity of the selected primers was verified by real-time PCR, melting curve analysis, and 2% agarose gel electrophoresis (Mupid-One, Eurogentec, USA). The 2% agarose gel was visualized under UV illumination using a ChemiDoc Imaging System (Bio-Rad, USA).

**Table 9:** Candidate reference genes and the target gene used in this study.

Gene (metabolic process)	Sequences of primers (5′–3′)	Amplicon length (bp)	Concentration of primers (nM)	References
*seb*	F: ACA CCC AAC GTT TTA GCA GAG AG R: CCA TCA AAC CAG TGA ATT TAC TCG	81	F: 200 R: 200	^63^
*16S rRNA* (translation)	F: TAT GGA GGA ACA CCA GTG GCG AAG R: TCA TCG TTT ACG GCG TGG ACT ACC	116	–	^64^
*fabD* (fatty acid biosynthesis)	F: CCT TTA GCA GTA TCT GGA CC R: GAA ACT TAG CAT CAC GCC	102	F: 200 R: 200	^26^
*ftsZ* (cell division)	F: TAT TAC TGG TGG CGA GTC A R: AGT ATT TAC GCT TGT TCG GA	223	F: 200 R: 200	^29^
*gmk* (nucleotide metabolism)	F: AAT CGT TTT ATC AGG ACC R: CTT CAC CTT CAC GCA TTT	120	F: 400 R: 400	^65^
*gyrB* (replication)	F: GTC GAA GGG GAC TCT G R: GCT CCA TCC ACA TCG G	242	F: 400 R: 400	^29^
*proC* (amino acid biosynthesis)	F: GGC AGG TAT TCC GAT TG R: CTT CCG GTG ATA GCT GTT A	231	F: 200 R: 200	^29^
*pyk* (glycolysis)	F: GCA TCT GTA CTC TTA CGT CC R: GGT GAC TCC AAG TGA AGA	89	–	^26^
*rho* (transcription)	F: GAA GCT GCT GAA GTC G R: CGT CCA TAC GTG AAC CC	319	F: 300 R: 300	^29^
*rpoB* (transcription)	F: CTA AGC ACA GAG GTC GT R: ACG GCA TCC TCA TAG T	298	F: 400 R: 400	^29^
*tpiA* (gluconeogenesis)	F: GGT GAA ACA GAC GAA GAG R: TTA CCA GTT CCG ATT GCC	145	F: 300 R: 300	^26^

Primer sequences are given in the 5′–3′ direction.

*F* forward primer, *R* reverse primer.

### qPCR

qPCR assays were performed using a LightCycler 480 II (Roche Life Science, Germany). The 10-µL reaction mixture consisted of 5 µL of Fast SG qPCR Master Mix (EURx, Poland), 200–400 nM each primer (TIB MOLBIOL, Germany), 3.2–3.6 µL of nuclease-free water (EURx, Poland) and 1 µL of fivefold diluted cDNA. The following steps were implemented in the PCRs: a pre-incubation step (95 °C for 5 min), followed by 45 cycles of amplification (denaturation at 95 °C for 15 s, annealing at 60 °C for 15 s and extension at 72 °C for 15 s, with a single fluorescence measurement after each extension step). After the amplification step, melting curve analysis was performed (95 °C for 5 s, 65 °C for 60 s and then a slow increase in temperature to 97 °C with continuous fluorescence measurement). The melting curve analysis was carried out to exclude primer-dimer formation or nonspecific amplification. The specificity of amplification was confirmed by the presence of a single peak in the melting curve analysis.

In the qPCR experiments, optimal primer concentrations of reference genes (between 200 and 400 nM) were determined. For every candidate reference gene, standard curves were constructed. The studied cDNA was subjected to fivefold serial dilution (1:1, 1:5, 1:25, 1:125, 1:625, and 1:3125; each dilution was conducted in triplicate). Evaluation of gene expression was performed in triplicate for each fivefold-diluted cDNA sample. In each run of the experiment, a non-template control (NTC) was included. Additionally, to exclude genomic DNA contamination, 1 µL of a randomly selected RNA sample was used as a template.

### Analysis of reference gene expression stability

Eight reference genes were included in the analysis. Gene expression stability was measured using tools based on Microsoft Excel: BestKeeper^27^, geNorm^25^ and NormFinder^60^. Additionally, the results were also analysed by RefFinder software, which is available at https://www.heartcure.com.au/reffinder/^28^.

### Analysis of gene expression

Because different PCR efficiencies of the target gene and reference genes were observed, the Pfaffl model was applied in this study^61^. According to the Pfaffl model, the expression of a target gene is shown as a ratio (R) expressed by the following equation:

$$R = \frac{{\left( {E_{target} } \right)^{{\Delta Cp_{target} \left( {control - sample} \right)}} }}{{\left( {E_{ref} } \right)^{{\Delta Cp_{ref} \left( {control - sample} \right)}} }}$$

where *E*_target_ is the efficiency of real-time PCR of the target gene, *E*_ref_ is the efficiency of real-time PCR of a reference gene, *∆Cp*_*target*_ is the difference between the crossing points of the target gene (Cp value of the control sample minus that of a particular sample), and *∆Cp*_*ref*_ is the difference between the crossing points of the reference gene (Cp value of the control sample minus that of a particular sample)^61^.

In the presented calculations, the untreated control (reference cells kept in the dark) served as the calibrator (normalized to 1). The R values were log_2_ transformed and served as the fold change values. The R values were expressed as the mean of three independent biological replicates ± standard error of the mean (SEM). The statistical analysis was performed using the GraphPad Prism 8 program (GraphPad Software, Inc., CA, USA). The data were analysed using one-way analysis of variance (ANOVA) and Dunnett’s multiple comparisons test. A *p*-value < 0.05 indicated a significant difference.

### Assessment of antibiotic susceptibility profile after consecutive cycles of aPDI

In this experiment, four parallel conditions were tested: (1) control samples, where cells were not subjected to any treatment; (2) aPDI-treated samples using RB and green light; (3) aPDI-treated samples using NMB and red light; and (4) samples treated with sub-minimum inhibitory concentration (MIC) amounts of ciprofloxacin. Three independent biological cultures were tested for each of the four conditions. In each case, the cells were cultured overnight. Next, the cells were diluted to 0.5 McF standard, and in the case of the control samples, 50 µL of diluted bacterial culture was transferred to 5 mL of fresh TSB for overnight growth (37 °C, 150 rpm). This step was repeated through 15 consecutive cycles. In the case of the aPDI conditions, an overnight culture of *S. aureus* was diluted to 0.5 McF standard, and 1 µL of PS was added to 100 µL of bacterial culture to a final concentration of 0.25 µM (RB) or 5 µM (NMB). Samples were transferred into 96-well plates, covered with aluminium foil and incubated for 10 min (RB) or 15 min (NMB) in an incubator shaker (37 °C, 150 rpm). After the incubation process, plates were illuminated under the LED light source using appropriate irradiation conditions (green light λ_max_ = 515 nm, irradiance 150 mW/cm^2^, total fluence 1 J/cm^2^; red light λ_max_ = 632 nm, irradiance 234 mW/cm^2^, total fluence 16.25 J/cm^2^). After irradiation, 10-µL aliquots of the bacterial samples were transferred into sterile PBS to perform ten-fold serial dilution (10^–1^–10^–4^ in a 96-well plate) to assure that sublethal aPDI conditions were maintained. The remaining 90 µL of the sample was centrifuged (3 min, 10,000 rcf) and washed with 90 µL of sterile PBS. Cells were suspended in 50 µL of PBS and transferred into 5 mL of fresh TSB medium for overnight growth. This step was repeated 15 times (15 passages). In the case of ciprofloxacin treatment, overnight *S. aureus* cultures were diluted to 0.5 McF standard. Fifty microlitres of 0.5 McF bacterial culture was transferred into 5 mL of fresh TSB for overnight growth. A sub-MIC amount of ciprofloxacin (CIP, Sigma-Aldrich, Germany) was added to the bacterial culture. *S. aureus* cultures were passaged 15 times.

The *S. aureus* samples from the 1st, 5th, 10th and 15th cycles were tested for susceptibility to the following antibiotics: fusidic acid (FA, Sigma-Aldrich, Germany), gentamycin (GEN, Sigma-Aldrich, Germany), linezolid (LZD, Cayman Chemical, USA), mupirocin (MUP, Cayman Chemical, USA), trimethoprim/sulphamethoxazole (SXT, Sigma-Aldrich, Germany), and vancomycin (VAN, Sigma-Aldrich, Germany). MICs were determined by the microbroth dilution method according to the European Committee for Antimicrobial Susceptibility Testing (EUCAST)^62^.

## Supplementary information

Supplementary information

## Data Availability

The datasets generated during and/or analysed during the current study are available from the corresponding author on reasonable request.
