# Supplementary material for: Validation of stable reference genes in Staphylococcus aureus to study gene expression under photodynamic treatment: a case study of SEB virulence factor analysis
Source: Sci Rep. 2020 Oct 1;10:16354. doi: 10.1038/s41598-020-73409-1 (PMC7530716; doi:10.1038/s41598-020-73409-1)
Supplement: Supplementary file 1 — Supplementary information [file 41598_2020_73409_MOESM1_ESM.docx]

**Validation of stable reference genes in Staphylococcus aureus to study gene expression under photodynamic treatment: a case study of SEB virulence factor analysis.**

P. Ogonowska, J. Nakonieczna

Intercollegiate Faculty of Biotechnology, University of Gdansk and Medical University of Gdansk, Poland

**Corresponding author:**

Dr. Joanna Nakonieczna,

Intercollegiate Faculty of Biotechnology, University of Gdansk and Medical University of Gdansk,

Abrahama 58, 80-822 Gdansk, Poland

Email: [joanna.nakonieczna@biotech.ug.edu.pl](mailto:joanna.nakonieczna@biotech.ug.edu.pl)

Phone: +48 58 5236327

**Supplementary material**

**Supplementary Figure 1.** Light-dose dependent photodynamic inactivation of *S. aureus*. The following photoinactivation conditions were applied: (A) 0.25 µM rose bengal, 515 nm, 150 mW/cm^2^, (B) 5 µM new methylene blue, 632 nm, 234 mW/cm^2^. aPDI – antimicrobial photodynamic inactivation, L+ cells treated with light, without photosensitizer.

A

**
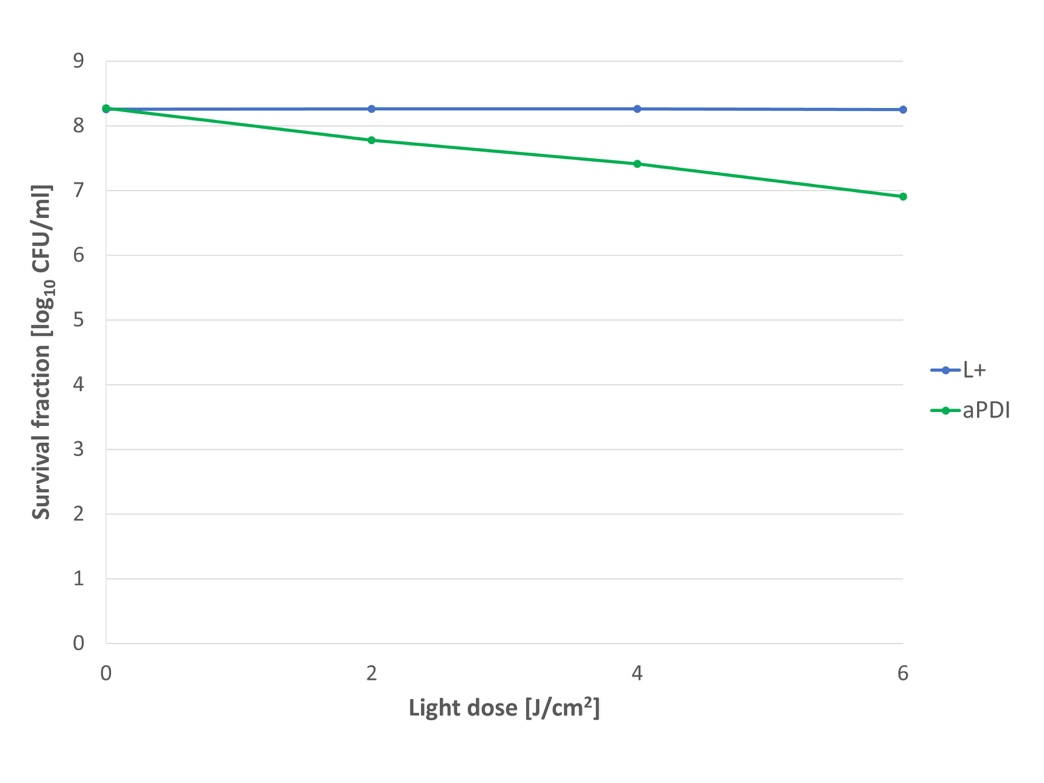

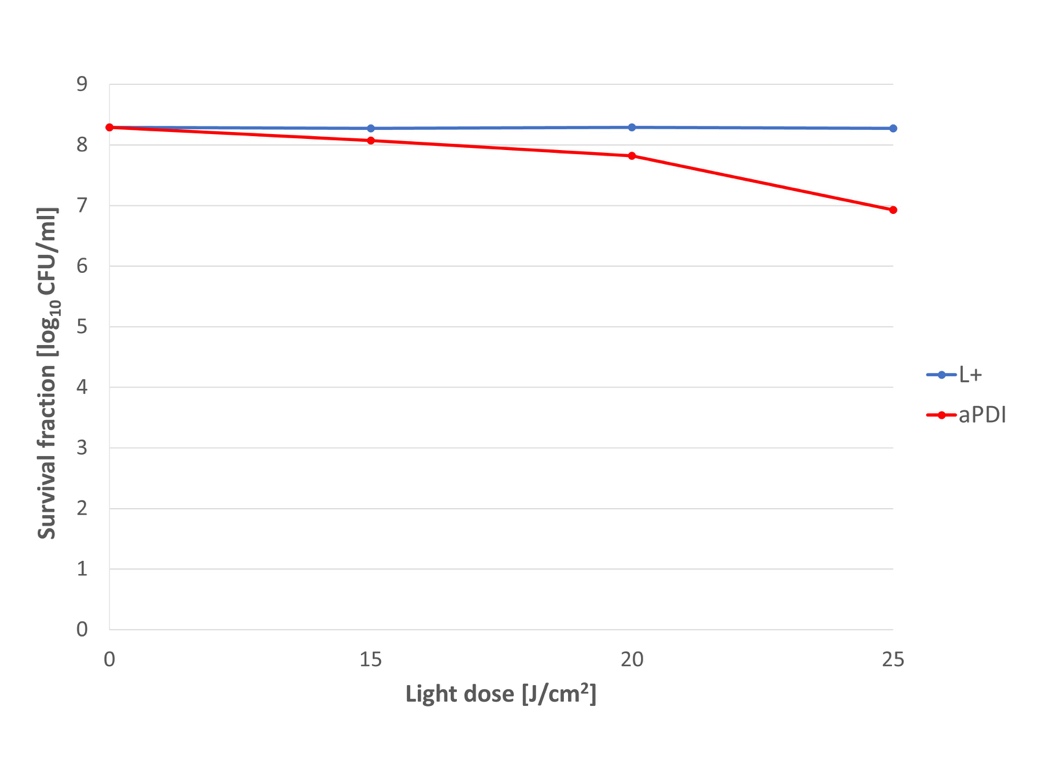
**

B

**Supplementary Figure 2.** Melting curves of candidate reference and the target genes tested. Melting curve analysis was performed under the following conditions: 95 °C for 5 seconds, 65 °C for 60 seconds, and then slowly increasing the temperature to 97 °C with continuous fluorescence measurement. The red line indicated the presence of a single peak for each of the tested genes, while the blue line indicated no template control (NTC). Single peaks shown indicate a single product and no non-specific amplification and primer-dimer formation. Light Cycler Instrument Software ver. 1.5; URL: www.roche-applied-science.com


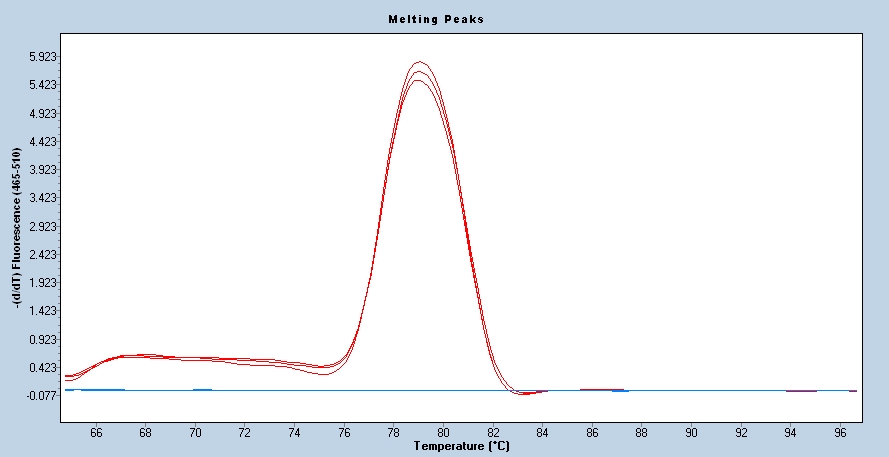


***seb***


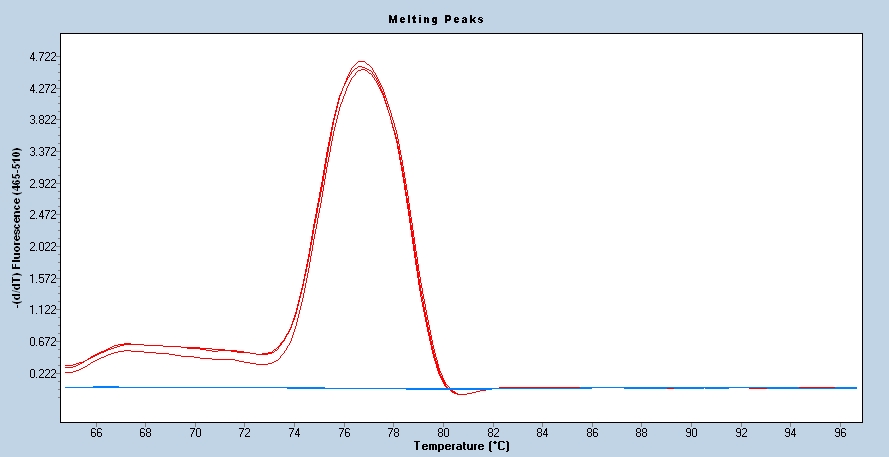


***fabD***


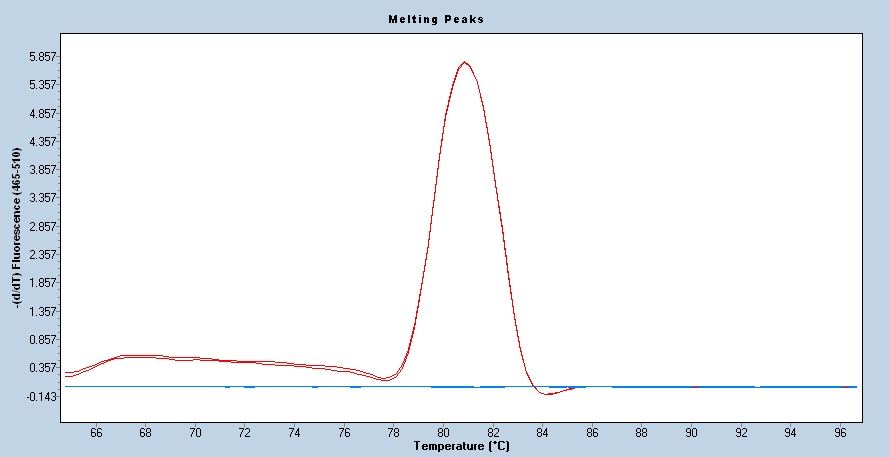


***ftsZ***


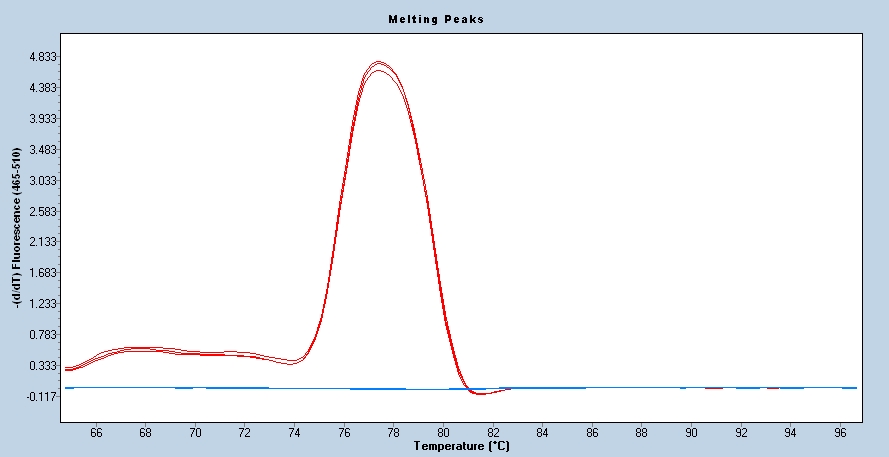


***gmk***


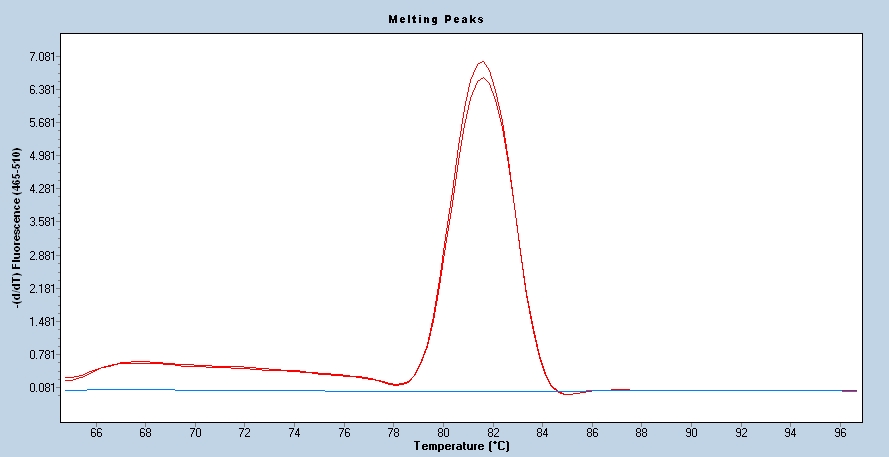


***gyrB***


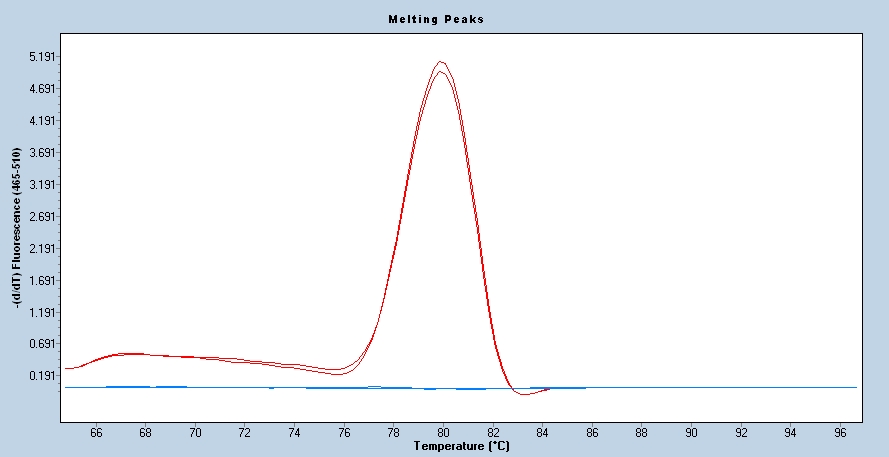


***proC***


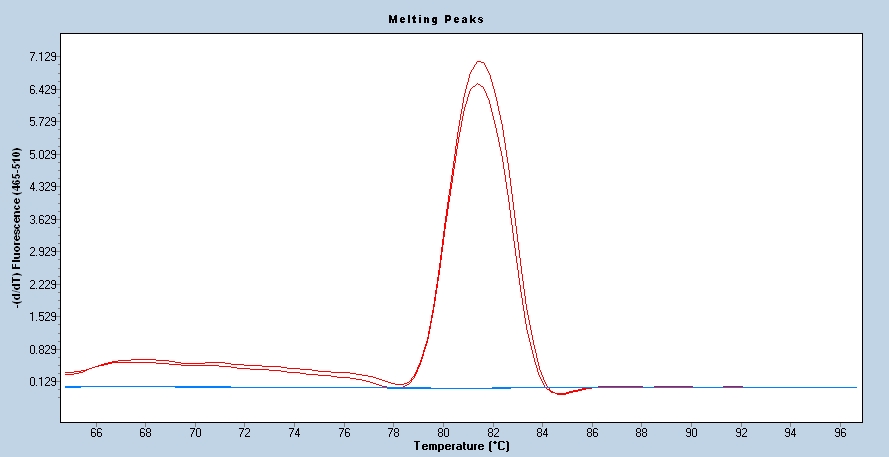


***rho***


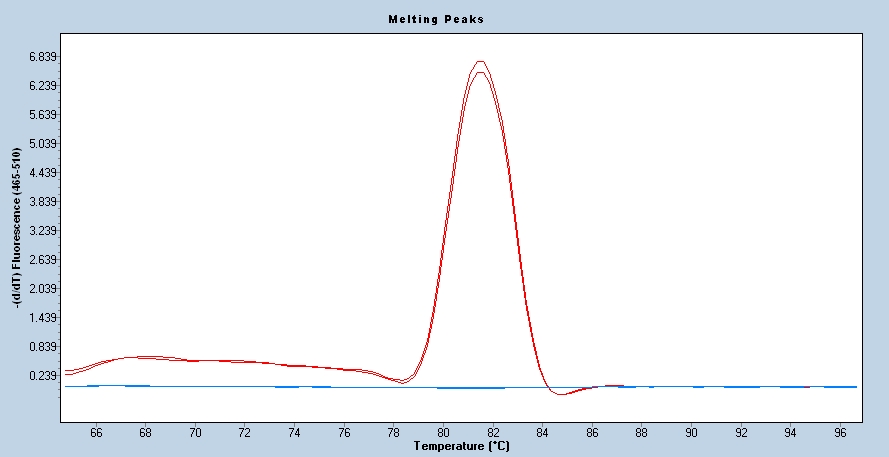


***rpoB***


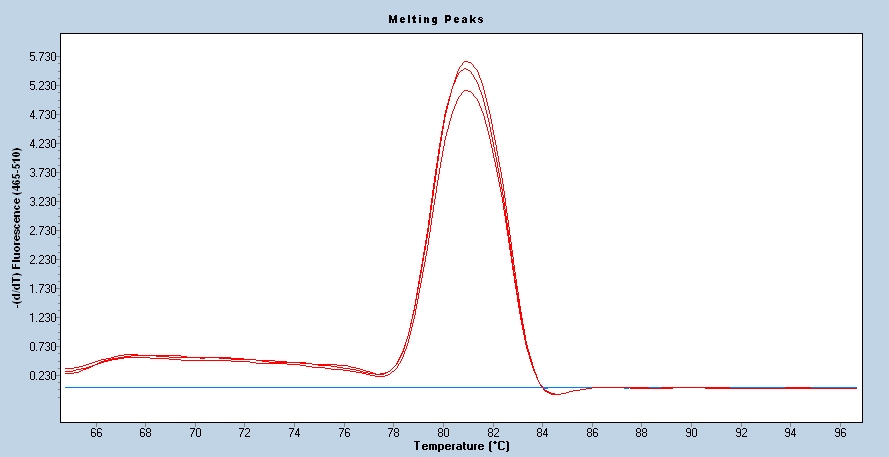


***tpiA***

**Supplementary Figure 3.** An exemplary electrophoretic separation result (2% agarose gel) showing a single qPCR products of the expected size for each candidate reference and target gene. 1. Marker*, 2. *seb* (81 bp), 3. *fabD* (102 bp)*,* 4. *ftsZ* (223 bp)*,* 5. *gmk* (120 bp)*,* 6. *gyrB* (242 bp)*,* 7. *proC* (231 bp)*,* 8. *rho* (319 bp)*,* 9. *rpoB* (298 bp)*,* 10. *tpiA* (145 bp)*,* 11. Marker*, bp – base pair

*GeneRuler 50bp DNA Ladder (Thermo Scientific)


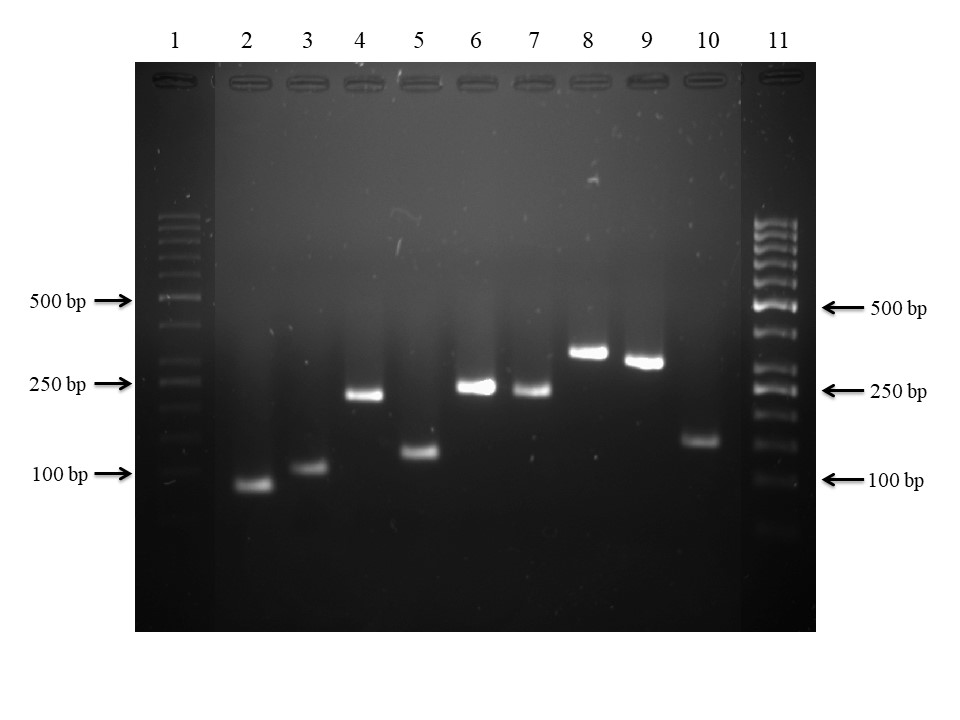


**Supplementary Figure 4.** Standard curves for qPCR primers. The tested cDNA was serially diluted 5-fold (1:1, 1:5, 1:25, 1:125, 1:625, 1:3125; each point in triplicate). Log Concentration – known standard cDNA concentrations, expressed in logarithm. Light Cycler Instrument Software ver. 1.5; URL: www.roche-applied-science.com


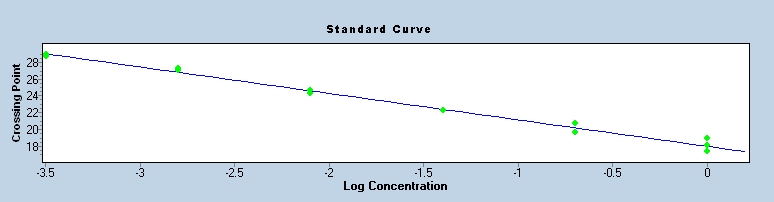


***seb***


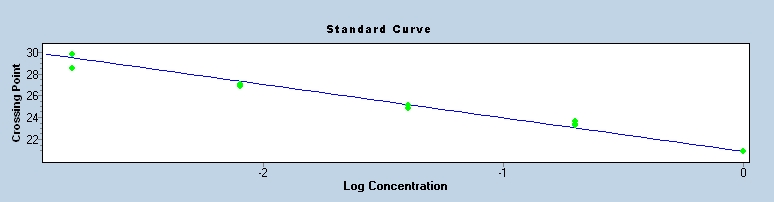


***fabD***


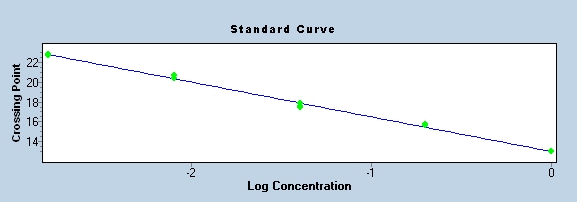


***ftsZ***


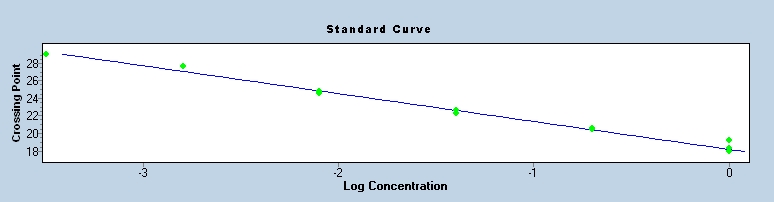


***gmk***


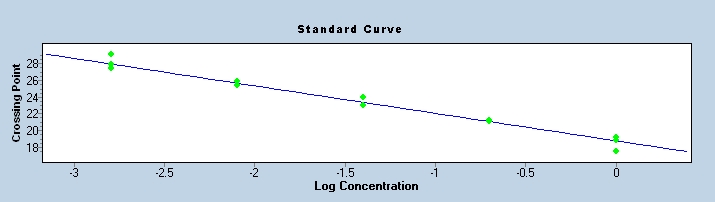


***gyrB***


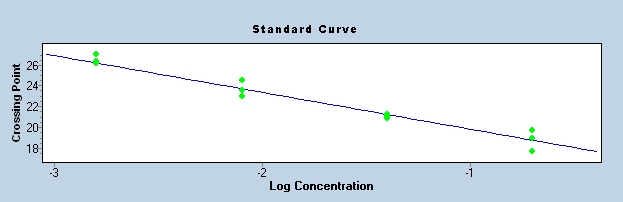


***proC***


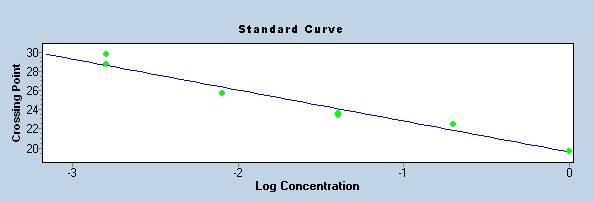


***rho***


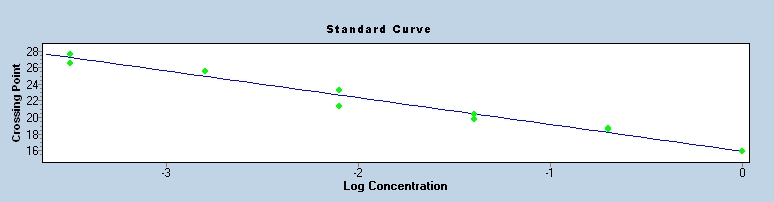


***rpoB***


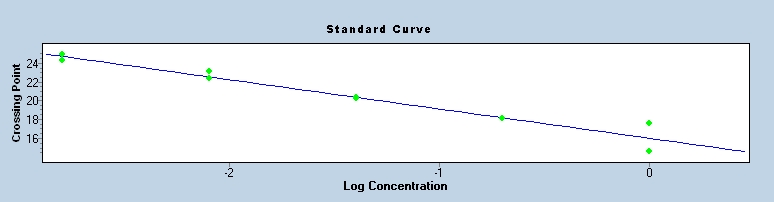


***tpiA***

**Supplementary Table 1.** Stability of the expression of eight selected candidate reference genes using geNorm and NormFinder for rose bengal and green light

| **Rose bengal and green light** | | | | | |
| --- | --- | --- | --- | --- | --- |
| **BestKeeper** | | **geNorm** | | **NormFinder** | |
| **Gene** | **r (*p*-value)** | **Gene** | **M value** | **Gene** | **Stability value** |
| *ftsZ* | 0.92 (0.001) | *ftsZ* | 1.236 | *ftsZ* | 0.092 |
| *rho* | 0.92 (0.001) | *gyrB* | 1.300 | *gyrB* | 0.135 |
| *tpiA* | 0.92 (0.001) | *gmk* | 1.318 | *gmk* | 0.146 |
| *gyrB* | 0.90 (0.001) | *rho* | 1.522 | *rho* | 0.191 |
| *gmk* | 0.86 (0.001) | *rpoB* | 1.575 | *proC* | 0.213 |
| *fabD* | 0.78 (0.001) | *fabD* | 1.614 | *fabD* | 0.229 |
| *rpoB* | 0.76 (0.001) | *proC* | 1.616 | *rpoB* | 0.236 |
| *proC* | 0.69 (0.001) | *tpiA* | 1.916 | *tpiA* | 0.336 |

**r –** Pearson correlation coefficient; ***p-*value –** probability; **M value –** the gene-stability measure

**Supplementary Table 2.** Stability of the expression of eight selected candidate reference genes using geNorm and NormFinder for new methylene blue and red light

| **New methylene blue and red light** | | | | | |
| --- | --- | --- | --- | --- | --- |
| **BestKeeper** | | **geNorm** | | **NormFinder** | |
| **Gene** | **r (*p*-value)** | **Gene** | **M value** | **Gene** | **Stability value** |
| *ftsZ* | 0.90 (0.001) | *ftsZ* | 1.056 | *ftsZ* | 0.085 |
| *rho* | 0.84 (0.001) | *proC* | 1.143 | *proC* | 0.135 |
| *tpiA* | 0.82 (0.001) | *fabD* | 1.230 | *fabD* | 0.149 |
| *proC* | 0.80 (0.001) | *rho* | 1.269 | *rho* | 0.180 |
| *fabD* | 0.76 (0.001) | *gyrB* | 1.324 | *gyrB* | 0.201 |
| *rpoB* | 0.75 (0.001) | *gmk* | 1.366 | *gmk* | 0.206 |
| *gmk* | 0.72 (0.001) | *rpoB* | 1.396 | *rpoB* | 0.216 |
| *gyrB* | 0.63 (0.001) | *tpiA* | 1.699 | *tpiA* | 0.290 |

**r –** Pearson correlation coefficient; ***p-*value –** probability; **M value –** the gene-stability measure
